# Interfacial Phenomena in Multi-Micro-/Nanolayered Polymer Coextrusion: A Review of Fundamental and Engineering Aspects

**DOI:** 10.3390/polym13030417

**Published:** 2021-01-28

**Authors:** Bo Lu, Huagui Zhang, Abderrahim Maazouz, Khalid Lamnawar

**Affiliations:** 1 Key Laboratory of Materials Processing and Mold (Ministry of Education), National Engineering Research Center for Advanced Polymer Processing Technology, Zhengzhou University, Zhengzhou 450002, China; bolu@zzu.edu.cn; 2 CNRS, UMR 5223, Ingénierie des Matériaux Polymères, INSA Lyon, Université de Lyon, F-69621 Villeurbanne, France; abderrahim.maazouz@insa-lyon.fr; 3 Fujian Key Laboratory of Polymer Science, College of Chemistry and Materials Science, Fujian Normal University, Fuzhou 350007, China; huagui.zhang@fjnu.edu.cn; 4 Hassan II Academy of Science and Technology, Rabat 10100, Morocco

**Keywords:** micro-/nanolayered polymers, interfacial phenomena, multilayer coextrusion

## Abstract

The multilayer coextrusion process is known to be a reliable technique for the continuous fabrication of high-performance micro-/nanolayered polymeric products. Using laminar flow conditions to combine polymer pairs, one can produce multilayer films and composites with a large number of interfaces at the polymer-polymer boundary. Interfacial phenomena, including interlayer diffusion, interlayer reaction, interfacial instabilities, and interfacial geometrical confinement, are always present during multilayer coextrusion depending on the processed polymers. They are critical in defining the microstructural development and resulting macroscopic properties of multilayered products. This paper, therefore, presents a comprehensive review of these interfacial phenomena and illustrates systematically how these phenomena develop and influence the resulting physicochemical properties. This review will promote the understanding of interfacial evolution in the micro-/nanolayer coextrusion process while enabling the better control of the microstructure and end use properties.

## 1. Introduction

Polymer blends and multilayers account for a very large fraction of high-performance materials used in industry today, because they are easy and cheap to prepare. In these multiphase polymer systems, interfacial phenomena such as interdiffusion, interfacial slippage, and/or interfacial reactions occurring at the polymer–polymer interface are of high interest for researchers in polymer science [1,2,3]. For instance, the interdiffusion process that occurs in the region between polymers plays a very important role in mixing and homogenizing composition gradients during processing [4,5]. Interfacial slippage may also occur in incompatible polymer systems, a phenomenon that has been suggested to explain the negative deviation in viscosity [3]. Nevertheless, it is undesirable due to its detrimental effect on the final properties of products, such as reduced adhesion between polymers. To improve this weak polymer–polymer adhesion in an incompatible system, a compatibilization effect is often introduced by adding premade copolymers as compatibilizers or by locally incorporating chemical reactions at the polymer–polymer interface [6,7]. Hence, an interphase that is a nonzero thickness 3D zone, as opposed to a purely geometrical plane interface, could be triggered either by interdiffusion or by chemical reaction at the polymer–polymer interfacial zone in a multiphase system. So far, the rheology, physics, morphological, and geometrical properties associated with such an interphase, as well its contribution to the multiphase blends and to multilayer systems, persist as a hot issue among polymer scientists and engineers.

**Figure 1:**
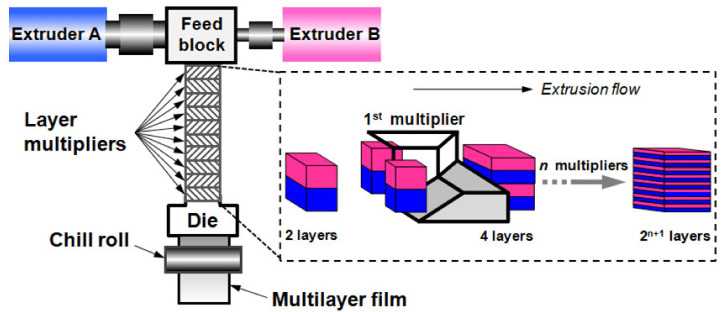
Schematic illustration of the multilayer coextrusion process. Reproduced with permission from Ref. [13]. Copyright (2020) John Wiley and Sons.

More specifically, for multilayered polymers that are widely used multiphase materials, polymer–polymer interfaces play a crucial role in the resulting properties. Generally, there are many techniques for combining different polymers to create multilayered films, including layer-by-layer assembly (LbL) [8], multilayer coextrusion [9], lamination, solvent casting, and spin coating. One of the most appealing techniques for industry is the coextrusion process, which is widely used to form multilayered sheets or films that are suitable for various products ranging from food packaging materials to dielectric capacitors to reflective polarizers [10,11,12]. In contrast to the LbL method, multilayer coextrusion is known as a forced-assembly concept in which two or more polymers are extruded by two or more extruders and combined in a feedblock or die to form a finished product with multiple layers (Figure 1) [5,13]. Notably, as opposed to traditional approaches using LbL assembly and spin coating that suffer from low productivity, this technology is a top-down approach that enables the industrial manufacture of multilayered films. Multilayer coextrusion is also a good tool for fundamental studies, especially for studying interfacial dynamics in polymer blending, since the morphology of coextruded multilayers is well defined by layer number and layer thicknesses, making this a suitable model system for multiphase systems [3,14]. When it comes to the processing problems posed by coextrusion, the subject is very vast. Researchers have dealt with many aspects ranging from equipment designs [15], fluid mechanics, and analysis of multilayer flow [16,17] to interfacial defects and optimization of processing conditions [18,19], etc. It is known that under certain operating conditions, interfacial defects can be observed inside the die, especially in the case of coextruded polymers with a large rheological contrast. These defects can be divided into two common types: one featuring a waviness and/or rugged shape at the polymer–polymer interface (i.e., interfacial flow instability) and another characterized by nonuniformity in layer thicknesses (i.e., encapsulation) [12]. The important theoretical and experimental advances made in the last few decades with regard to such interfacial defects have been mainly limited to mechanical and numerical approaches.

In addition, with the rapid development of coextrusion processing technology, the nanolayer coextrusion technique invented by incorporating the concept of layer multiplication has made much progress in the past two decades [20,21]. Using layer multipliers or layer-multiplying elements, products with thousands of layers can be produced, in which the layer thickness can be reduced to the nanometer scale (Figure 1) [22,23]. Interfacial spatial confinement always dominates when layer thickness is decreased, which greatly alters the microstructure and dynamics of the multilayer polymers [24,25]. Macroscopic properties, including mechanical, electric, and gas/liquid barrier properties, are also dramatically affected. In addition, for the nanolayer coextrusion process, the laminar flow conditions combine polymers in the layer multipliers by producing a large number of interlayer interfaces without complete mixing [5]. Interfacial behaviors involving interlayer diffusion and reactions are also critical in defining the structure and properties [5,13]. The measurement of the interfacial properties is important for understanding the interdependence of processing, structure, and properties.

Therefore, this paper provides an overview of the research work carried out in academia in recent years regarding interfacial phenomena, notably interlayer diffusion, interlayer reaction, interfacial instabilities, and interfacial geometrical confinement. The effects of these phenomena on microstructural development and microscopic properties have been summarized. Relevant basic theories and experimental techniques are also included. This review will enable a better understanding of the interfacial phenomena involved in multilayer coextrusion, in view of establishing the processing-structure-property relationship of multi-micro-/nanolayered polymer products.

## 2. Interfacial Phenomena at Polymer–Polymer Interfaces

### 2.1. Interfacial Diffusion

When two polymers are brought into contact at a temperature above the glass transition temperature, particularly at the melt state, the movement of polymer chains across the interface results in an interdiffusion process driven by the entropic advantage between components [26,27,28,29]. Interdiffusion has been an important issue in multicomponent polymer blends and multilayered composites. The presence of interdiffusion enhances polymer–polymer adhesion and stabilizes the interfaces [26,30,31]. This phenomenon is also important in controlling the glass transition temperatures [32,33], microstructure development [5,34], gas permeability [35], and mechanical [30], optical [4], and electric properties [36] of multicomponent polymer products.

#### 2.1.1. Basic Interdiffusion Theories

During the diffusion process, the rate of disappearance of the concentration gradient across the interface, referred to as the mutual diffusion coefficient (*D_m_*), is dependent on the composition of the system, as well as the excess enthalpy and entropy of segment–segment mixing:

(1) $$D_{m}=2(\chi_{s}-\chi )\varphi_{A}\varphi_{B}D_{T}=\left(\frac{\varphi_{B}}{N_{A}}+\frac{\varphi_{A}}{N_{B}}-2\chi \varphi_{A}\varphi_{B}\right)D_{T},$$

where $\chi_{s}$ is the interaction parameter at the spinodal of the A/B mixture:

(2) $$\chi_{s}=\frac{1}{2}\left(\frac{1}{\varphi_{A}N_{A}}+\frac{1}{\varphi_{B}N_{B}}\right).$$

*D_T_* is the transport coefficient related to the mobility of segments or the monomeric friction coefficient of the components involved [37]. Two different theories concerning *D_T_* have been proposed, including the fast-mode theory with a faster moving species dominating the diffusion mechanism [38] and the slow-mode theory with a slower moving species [39]. Both theories were originated from the Flory–Huggins Lattice theory via Onsager formalism. The driving force for interdiffusion is the chemical potential gradient.

The flux $J_{i}$ is related to the chemical potential $\mu_{i}$ as follows:

(3) $$J_{A}=-\Lambda_{A}\nabla \left(\mu_{A}-\mu_{V}\right)$$

(4) $$J_{B}=-\Lambda_{B}\nabla \left(\mu_{B}-\mu_{V}\right)$$

(5) $$J_{V}=\Lambda_{A}\nabla \left(\mu_{A}-\mu_{V}\right)+\Lambda_{B}\nabla \left(\mu_{B}-\mu_{V}\right).$$

where $\Lambda_{i}$ is the Onsager coefficient of lattice *i* and subscripts *A, B,* and *V* denote molecule A, molecule B, and vacancy, respectively.

The chemical potential gradient is obtained from the Flory–Huggins theory:

(6) $$\nabla \mu_{i}=\frac{k_{B}T}{\varphi_{i}}\left(\frac{\varphi_{B}}{N_{A}}+\frac{\varphi_{A}}{N_{B}}-2\chi \varphi_{A}\varphi_{B}\right)\nabla \varphi_{i},$$

where *k_B_* is the Boltzmann constant and *T* is the temperature.

The slow-mode theory assumes no vacancy flux (*J_V_* = 0), leading to:

(7) $$J_{A}=-J_{B}=\frac{-\Lambda_{A}\Lambda_{B}}{\Lambda_{A}+\Lambda_{B}}\nabla \left(\mu_{A}-\mu_{B}\right).$$

Combining Equations (5)–(7) of continuity for component *A*:

(8) $$\frac{1}{\Omega }\frac{\partial \varphi }{\partial t}=\nabla \left(-J_{A}\right)$$

yields:

(9) $$\frac{\partial \varphi }{\partial t}=\nabla \left(D_{m}\nabla \varphi \right)=\nabla \left(-\Omega J_{A}\right)=\nabla \left[\frac{\Omega k_{B}T}{\varphi_{A}\varphi_{B}}\left(\frac{\Lambda_{A}\Lambda_{B}}{\Lambda_{A}+\Lambda_{B}}\right)\left(\frac{\varphi_{B}}{N_{A}}+\frac{\varphi_{A}}{N_{B}}-2\chi \varphi_{A}\varphi_{B}\right)\nabla \varphi_{i}\right]$$

and *D_m_* can be thus obtained:

(10) $$D_{m}=\frac{\Omega k_{B}T}{\varphi_{A}\varphi_{B}}\left(\frac{\Lambda_{A}\Lambda_{B}}{\Lambda_{A}+\Lambda_{B}}\right)\left(\frac{\varphi_{B}}{N_{A}}+\frac{\varphi_{A}}{N_{B}}-2\chi \varphi_{A}\varphi_{B}\right),$$

where Ω is the volume of a quasi-lattice site.

On the contrary, the fast-mode theory assumes $J_{V}\neq 0$ but rather $\nabla \mu_{V}=0$, which leads to the total flux $J_{A}^{T}$ of A:

(11) $$J_{A}^{T}=-\Lambda_{A}\varphi_{B}\nabla \mu_{A}+\Lambda_{B}\varphi_{A}\nabla \mu_{B}.$$

Similarly, an expression of *D_m_* can be given:

(12) $$D_{m}=\Omega k_{B}T\left(\frac{\varphi_{B}}{\varphi_{A}}\Lambda_{A}+\frac{\varphi_{A}}{\varphi_{B}}\Lambda_{B}\right)\left(\frac{\varphi_{B}}{N_{A}}+\frac{\varphi_{A}}{N_{B}}-2\chi \varphi_{A}\varphi_{B}\right).$$

Comparing Equation (13) with Equation (1) and making some rearrangements, we can obtain:

(13) $$D_{T}=\varphi_{B}N_{A}D_{A}^{*}+\varphi_{A}N_{B}D_{B}^{*},$$

with the tracer diffusion coefficient of component *i*, $D_{i}^{*}$, as:

(14) $$D_{i}^{*}=\frac{\Omega k_{B}T}{N_{i}\varphi_{i}}\Lambda_{i}.$$

The Onsager coefficient ($\Lambda_{i}$) is then extracted in terms of the curvilinear Rouse mobility ($B_{0,i}$) or in terms of the monomeric friction coefficient ($\zeta_{i}$) of the segment *i*:

(15) $$\Lambda_{i}=\frac{B_{0,i}N_{i}^{e}}{\Omega N_{i}}\varphi_{i}=\frac{N_{i}^{e}}{\zeta_{i}\Omega N_{i}}\varphi_{i},$$

where $N_{i}^{e}$ is the number of repeat units per entanglement length of component *i*, respectively.

The slow-mode theory assumes the equal and opposite fluxes of the two polymers, indicating that the interface remains symmetrical as interdiffusion proceeds. By contrast, the fast-mode theory describes the interdiffusion with a moving interface by unequal fluxes that are balanced by a net flux of vacancies across the interface. It suggests a movement of the interface toward the more quickly diffusing component and a broadening of the concentration profile on the slower component side.

#### 2.1.2. Interdiffusion in Micro-/Nanolayer Coextrusion

**Figure 2:**
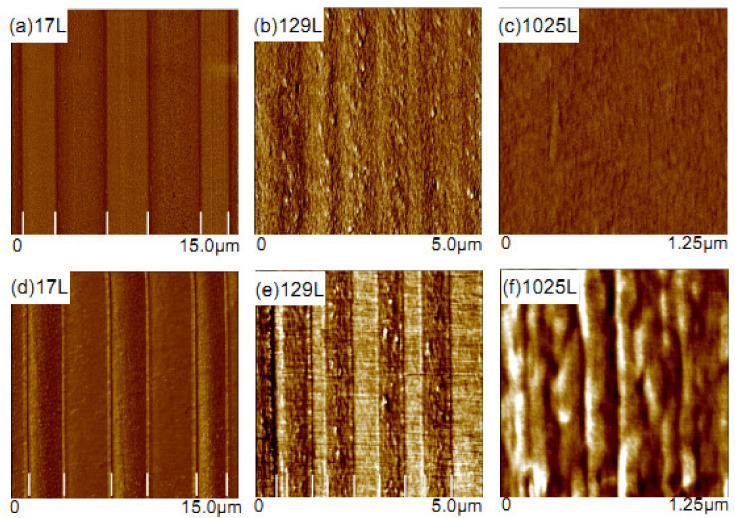
Atomic force microscope (AFM) phase images showing the layer morphology in multilayer films with a varying number of layers for (**a**–**c**) nylon/ethylene vinyl alcohol (44 mol% ethylene) (EVOH44) multilayers and (**d**–**f**) nylon/24 mol% ethylene (EVOH24) multilayers. (**a**,**d**) It shows 17 layers with nominal layer thickness of 3.2 µm; (**b**,**e**) 129 layers with nominal layer thickness of 400 nm; and (**c**,**f**) 1025 layers with nominal layer thickness of 50 nm. Reproduced with permission from Ref. [40]. Copyright (2016) Elsevier.

Interdiffusion also occurs in multilayer coextrusion, in which a resulting interphase is generated by localized interlayer mixing [4,35,36]. This interdiffusion behavior in multilayer coextrusion can be viewed in Figure 2, which illustrates the coextrusion of nylon with two different grades of ethylene vinyl alcohol (EVOH) with different ethylene contents: EVOH44 (44 mol% ethylene) and EVOH24 (24 mol% ethylene) [40]. Obviously, well-defined layer boundaries with sharp interfaces can be observed in 17-layered films, and 129-layered films show a diffused interphase, but the two polymer components are still visible in this sample. When the number of layers increases to 1025, however, completely interdiffused nylon/EVOH layers are observed therein. This clearly indicates the existence of interdiffusion during coextrusion due to the intermixing of component layers. Miscibility between the nylon and EVOH melts causes the layers to diffuse from the time that they are combined in the feedblock until the melt is quenched after exiting the die. Coextrusion through an increasing number of layer-multiplying dies increases the number of layer interfaces and prolongs the melt contact time while decreasing the layer thicknesses. The degree of interlayer interdiffusion rapidly increases and, after several multiplications, forces the entire melt toward a homogeneous blend.

A few studies have addressed theories describing the interdiffusion phenomenon during practical coextrusion processing. In a pioneering work, Baer and his coworkers studied interdiffusion in a microlayered system of polycarbonate (PC) and copolyester (KODAR) and proposed an interdiffusion model to explain the diffusion occurring during the coextrusion process [41]. An interfacial composition profile can be calculated via Fick’s equation as follows:

(16) $$\frac{\partial W_{i}}{\partial t}=D_{m}\frac{\partial^{2}W_{i}}{\partial x^{2}},$$

where *W_i_* is the weight fraction of species *i*, *t* is the diffusion time, *x* is the position, and *D_m_* is the mutual diffusion coefficient. Only one-half layers of the two components were considered due to the symmetry of the layered structure. Many assumptions equivalent to the case of weakly interacting polymer pairs were used in this analysis. There are some assumptions: (i) the mutual diffusion coefficient is dependent on temperature but independent of the composition; (ii) the position of the interface between adjacent layers is constant; and (iii) the initial interface is sharp, and the composition gradient at the boundaries of the interdiffusion element is equal to zero:

(17) $${\left(\frac{\partial W_{PC}}{\partial t}\right)}_{x=0}={\left(\frac{\partial W_{PC}}{\partial t}\right)}_{x=(L_{PC}+L_{k})/2}=0.$$

On the basis of these assumptions, this equation can be solved by the method of separation of variables as follows:

(18) $$\begin{array}{l} W_{PC}(x,t)=\frac{L_{PC}}{L_{PC}+L_{K}}+\sum_{n=1}^{\infty }\frac{2}{n\pi }\sin\left(\frac{n\pi L_{PC}}{L_{PC}+L_{K}}\right)\\ \;\;\;\;\;\;\;\;\;\;\;\;\;\;\;\;\times \cos\left(\frac{2\pi nx}{L_{PC}+L_{K}}\right)\exp\left(-\frac{4\pi^{2}n^{2}D_{m}t}{{\left(L_{PC}+L_{K}\right)}^{2}}\right) \end{array}$$

where *L_PC_* and *L_K_* are the layer thicknesses of PC and KODAR, respectively. By this equation, the concentration profiles across the layers can be mapped by relating the mutual diffusion coefficient to the gas permeability.

Note that the above interdiffusion model proposed by Baer and his coworkers follows a slow-model diffusion mechanism that assumes the mutual diffusion coefficient to be independent of the composition. It, therefore, might be unable to describe fast-controlled diffusion, especially for cases of diffusion in which the mutual diffusion coefficient is strongly dependent on the composition [42]. Very recently, Lamnawar and coworkers proposed a modified rheological model from a primitive Qiu–Bousmina model [42] to determine the mutual diffusion coefficient (*D_m_*) with composition dependence and subsequently mapped the interfacial diffusion profile in coextruded layers based on polymer dynamics theory and fast-controlled mode theory [43,44,45,46]. Assuming an apparent friction coefficient ($\zeta_{b}$) for the chain mixture at the diffuse interphase, the Onsager coefficient ($\Lambda_{i}$) takes the following form:

(19) $$\Lambda_{i}=\frac{N_{b}^{e}}{\zeta_{b}\Omega N_{i}}\varphi_{i}.$$

where $N_{b}^{e}$ is the average number of repeat units between entanglements for the polymer pair, and $\zeta_{b}$ is strongly composition dependent [43,47]. Thus, the mutual diffusion coefficient (*D_m_*) can be related to the structural properties of the mixture of A and B:

(20) $$D_{m}=\frac{k_{B}TN_{b}^{e}}{\zeta_{b}}\left(\frac{\varphi_{B}}{N_{A}}+\frac{\varphi_{A}}{N_{B}}\right)\left(\frac{\varphi_{B}}{N_{A}}+\frac{\varphi_{A}}{N_{B}}-2\chi \varphi_{A}\varphi_{B}\right).$$

The mutual diffusion coefficient (*D_m_*) can be experimentally quantified by its relationship with the rheological behavior of the interphase using a planar polymer A/B sandwich with healing time:

(21) $$\frac{1}{G_{I}^{*}(t)}=\frac{H}{2{\left(D_{m}t\right)}^{1/2}}\left(\frac{1}{G_{s,t}^{*}}-\frac{1}{G_{s,0}^{*}}\right)+\left(\frac{\varphi_{A}}{G_{A,0}^{*}}+\frac{\varphi_{B}}{G_{B,0}^{*}}\right),$$

where $G_{I}^{*}(t)$ is the complex modulus of the interphase at the healing time *t*; *H* is the total thickness of sandwich assembly; $G_{s,t}^{*}$ and $G_{s,0}^{*}$ are the overall complex moduli of sandwich assembly at healing times of *t* and 0, respectively; and $G_{A,0}^{*}$ and $G_{B,0}^{*}$ are the complex moduli of polymers A and B at healing time *t* = 0, respectively. *D_m_* can be related to the monomeric friction coefficient ($\zeta_{b}$) for the mixture as follows:

(22) $$\zeta_{b}=\frac{\pi^{2}k_{B}e_{b}{}^{2}T}{N_{b}{}^{3}b^{4}}\frac{1}{\omega }{\left[{\left(\frac{8G_{N,b}^{0}}{\pi^{2}G_{I}^{*}(t)}\right)}^{2}-1\right]}^{-1/2},$$

where *e_b_* represents the stem length on the order of the gyration radius of entanglements, *N_b_* is the number of repeat units of the blend, *b* is the effective bond length, *ω* is the angular frequency, and $G_{N,b}^{0}$ represents the average plateau modulus of the interphase/blend. The mutual diffusion coefficient can be calculated using:

(23) $$D_{m}={\left[\frac{{\left(2/3\right)}^{1/3}p}{{\left(9q+\sqrt{3}\times \sqrt{-4p^{3}+27q^{2}}\right)}^{1/3}}+\frac{{\left(9q+\sqrt{3}\times \sqrt{-4p^{3}+27q^{2}}\right)}^{1/3}}{2^{1/3}\times 3^{2/3}}\right]}^{2}$$

with

(24) $$p=\frac{8\delta \omega G_{N,b}^{0}}{\pi^{2}}\left(\frac{\varphi_{A}}{G_{A,0}^{*}}+\frac{\varphi_{B}}{G_{B,0}^{*}}\right)$$

(25) $$q=\frac{8\delta \omega G_{N,b}^{0}}{\pi^{2}}\frac{H}{2t^{1/2}}\left(\frac{1}{G_{s,t}^{*}}-\frac{1}{G_{s,0}^{*}}\right)$$

(26) $$\delta =\frac{N_{b}^{e}N_{b}{}^{3}b_{b}{}^{4}}{\pi^{2}e_{b}{}^{2}}\left(\frac{\varphi_{B}}{N_{A}}+\frac{\varphi_{A}}{N_{B}}\right)\left(\frac{\varphi_{B}}{N_{A}}+\frac{\varphi_{A}}{N_{B}}-2\chi \varphi_{A}\varphi_{B}\right).$$

The resultant interlayer interphase thickness can be also estimated according to:

(27) $$h_{I}^{\prime }=2{\left(D_{m}t\right)}^{1/2}.$$

The concentration profile of diffusing species across the interlayer interface can be also approximated by the simple Fickian solution:

(28) $$\varphi (z,t)=\frac{1}{2}\left[erf\left(\frac{h-z}{2{\left(D_{m}t\right)}^{1/2}}\right)+erf\left(\frac{h+z}{2{\left(D_{m}t\right)}^{1/2}}\right)\right],$$

where *erf* is the error function, *h* is the layer thickness, and *z* is the spatial axis along the diffusion direction with the boundary of layers as *z* = 0.

**Figure 3:**
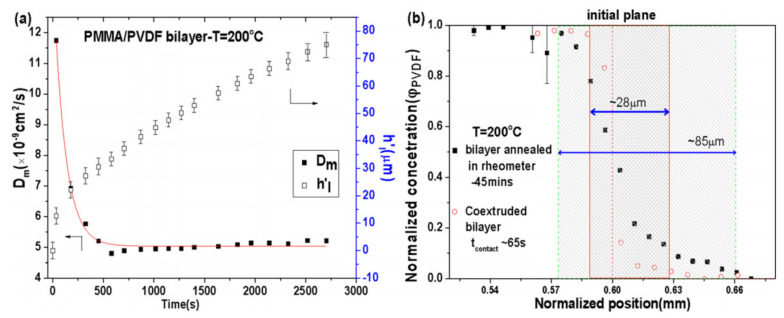
(**a**) Time evolution of the mutual diffusion coefficient and the interphase thickness determined from rheology with a poly(methyl methacrylate) (PMMA)/poly(vinylidene fluoride) (PVDF) bilayer healed at 200 °C, (**b**) normalized PVDF concentration profile versus normalized position determined from SEM-energy-dispersive X-ray analysis (EDX) in the cross-section of the bilayer after healing for 45 min (black solid squares) and the coextruded bilayer (red open circles). The sparse and dense shadow zones designate the interphase scale in the healed and coextruded bilayer, respectively. Reproduced with permission from Ref. [45]. Copyright (2016) AIP Publishing.

**Figure 4:**
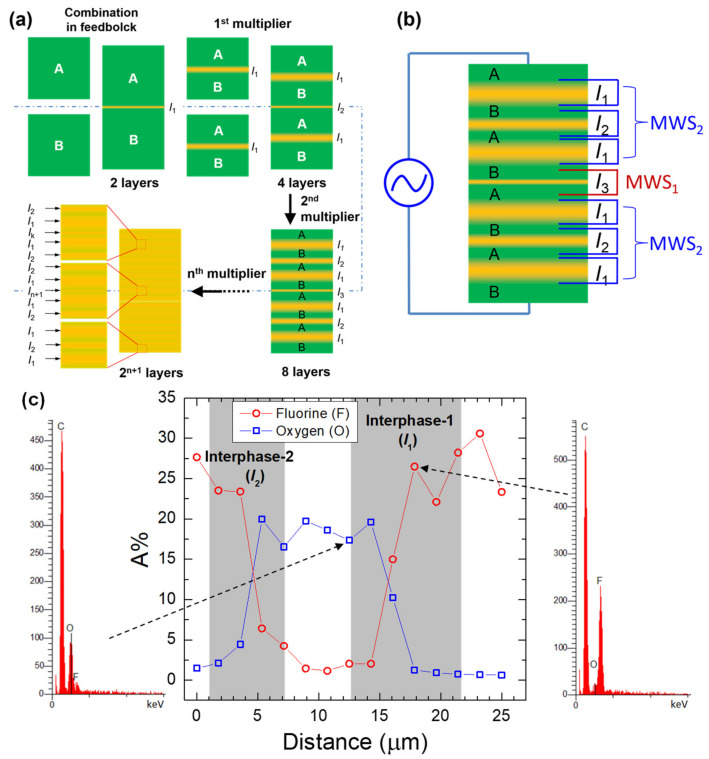
(**a**) A schematic illustration showing the interdiffusion and development of diffuse interphases during the coextrusion process for a compatible multilayered A/B system. “*I*” stands for the interphase shown by the bright regions in the schematic layered structures. (**b**) Scheme explaining the bimodal interfacial Maxwell–Wagner–Sillars (MWS) relaxations measured by dielectric relaxation spectroscopy for multilayered films subjected to an alternating electric field. (**c**) EDX measured concentration profile of F and O (in atomic fraction) versus measured positions for an eight-layer PVDF/PMMA film. Reproduced with permission from Ref. [5]. Copyright (2018) American Chemical Society.

The interdiffusion model above makes it possible to map the time evolution of the interphase profile including the mutual diffusion coefficient, interphase thickness, and concentration profile of diffusing species across the interface. Figure 3 shows an example of the quantified evolution of the mutual diffusion coefficient and the interphase thickness determined from rheological modeling for a compatible poly(vinylidene fluoride) (PVDF)/poly(methyl methacrylate) (PMMA) bilayered system, as well as the concentration profile determined from dispersive X-ray analysis (EDX) in the cross-section of the healed bilayer and the coextruded bilayer [45]. Notably, the calculated interphase thicknesses are in quantitative agreement with that determined by energy-dispersive X-ray analysis (EDX), indicating the validation of the interdiffusion model. In addition, as well documented in a recent study, the development of multiple interlayer interphases from interdiffusion and relevant length scales in nanolayered coextruded polymer films could be further quantitatively determined by the combination of dielectric relaxation spectroscopy and energy-dispersive X-ray analysis (Figure 4) [5].

### 2.2. Interfacial Instabilities

#### 2.2.1. Interfacial Flow Instabilities

Interfacial instability is an unstable process in which the interface between neighboring layers changes locally or loses its continuity. Interfacial distortion by flow instability can cause nonuniform layer thicknesses, irregular interfaces, and even nonuniform film thickness. Other interfacial flow instabilities include zigzag and wave-type in the extrudate [48,49]. A zigzag is observed along the flow direction, which is triggered in the die land above the critical interfacial shear stress [50]. In addition, a layer breakup phenomenon appears during coextrusion when the thicknesses of layers are reduced to beneath a critical value [51]. Generally, these interfacial instabilities can be reduced or eliminated by controlling the shear stress, extrusion rates, die gap, feedblock design, polymer viscoelasticity, etc.

#### 2.2.2. Interfacial Slip

Most commercial polymers are immiscible, and the interfacial region between them may be of a lower density than the constituent bulk materials. A significant slip can, therefore, occur during the flow due to reduced entanglements at their interface [52]. Especially for the cases in which components in a layered material have little compatibility or adhesion, densitometry variations within layered films with high numbers of layer interfaces can result in nontrivial interphase volumes. Interphases with reduced polymer chain entanglements have been demonstrated to induce interfacial instability, when films are processed under high shear rates through a mechanism assigned to the interfacial slip.

**Figure 5:**
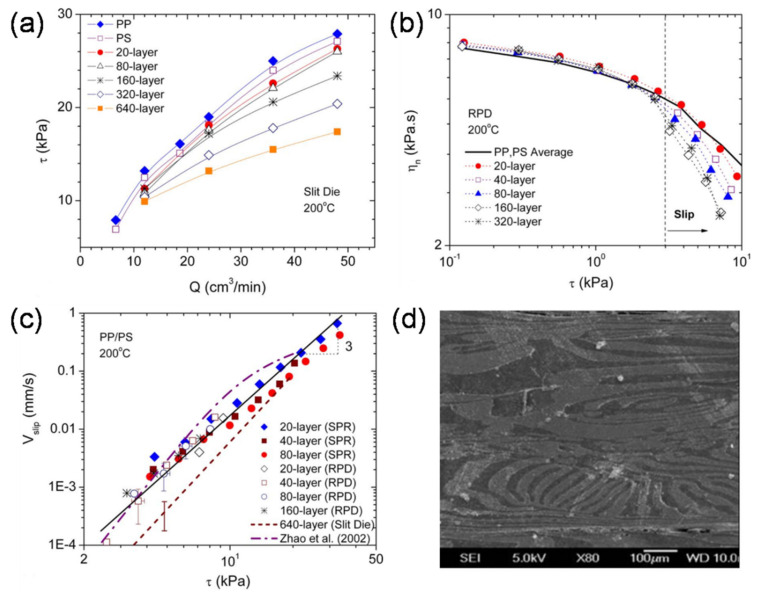
(**a**) Pressure drop of multilayered polypropylene (PP)/polystyrene (PS) samples and neat components in an in-line slit rheometer; (**b**) nominal viscosity of multilayer samples and harmonic average (solid lines) of the neat components, as measured by steady shear experiments with a rotational parallel-disk rheometer; (**c**) interfacial slip velocities of multilayers; and (**d**) SEM micrographs of 320-layer PP/PS multilayer samples after steady shear. Reproduced with permission from Ref. [53]. Copyright (2009) AIP Publishing.

Interfacial slip in multilayer systems was ever studied using coextruded polypropylene (PP)/polystyrene (PS) multilayers with closely matched viscosities [3,53,54]. An obvious deviation was found in the measured pressure drops of multilayers from those of the homopolymers as well as in the nominal viscosity, especially for larger numbers of layers (Figure 5) [53]. These deviations increased with the flow rate. These data are strong evidence for apparent slip, indicating a thin, low-viscosity layer between the PP and PS interfaces. Significant layer instability, characterized by layer breakup and/or delamination, was found in nanolayered samples, which could result from interfacial slip (Figure 5). Besides, with another multilayered system of PS/PMMA (smaller Flory–Huggins parameter *χ* than PP/PS), a slight pressure drop and slip velocity decreased with the interfacial width [3]. In addition, the incorporation of a premade polystyrene-block-ethyl-ethylene-diblock copolymer P(S-b-EE) to the PP/PS system could suppress the interfacial slip [3]. The interfacial slip phenomenon suggests the importance of interfacial interactions and adequate adhesion in multilayered polymers during processing, beyond the common expectation of matching viscosities. Eliminating the possibility of interfacial slip is of remarkable importance if highly regular and continuous layer architecture is required for the target properties.

#### 2.2.3. Layer Breakup in Nanolayer Coextrusion

**Figure 6:**
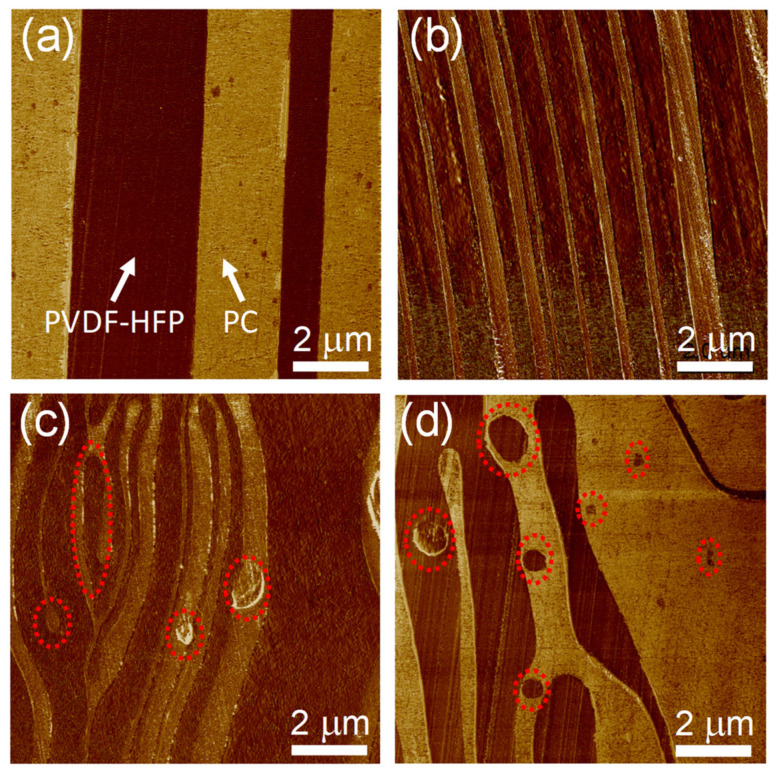
AFM phase images for cross-sections of poly(vinylidene fluoride-*co*-hexafluoropropylene) (PVDF-HFP)/polycarbonate (PC) films with various numbers of layers: (**a**) 32 L, (**b**) 256 L, (**c**) 2048 L, and (**d**) 16384 L.

The layer breakup phenomenon has been observed in many nanolayered systems during coextrusion, with layers breaking spontaneously during the process and losing their integrity [33,51,55,56,57]. This breakup phenomenon has been observed in different polymer pairs, and the layer-continuity limit appeared to be system dependent. Hiltner and coworkers found that efforts to obtain PMMA nanolayers thinner than 5 nm resulted in layer breakup and instability in polycarbonate (PC)/PMMA multilayers [33]. Meanwhile, in a PP/PS system, the layers break up when the layer thickness is thinner than 25 nm PP/PS [56]. Layer breakup alters the layered structure and can even adversely affect the final properties. In the presence of layer breakup, Hiltner and coworkers observed a reduction in the barrier property of PP/poly(ethylene oxide) (PEO) nanolayer film [58]. For the moment, the mechanism governing layer breakup in nanolayers is still lacking in the open literature. Interfacial distortions (viscous encapsulation or secondary flows) during coextrusion might be generally responsible for the layer breakup. In addition, interfacial instabilities due to an initial disturbance at the interfaces may be amplified along the flow in the die, which can also induce layer ruptures [59]. In addition, material characteristics have also been demonstrated to contribute to the layer breakup. With a PS/PMMA multilayer system, Bironeau et al. recently reported the existence of a critical layer thickness of around 10 nm, below which the layers break up [51]. They attributed this breakup phenomenon to small interfacial disturbances that are amplified by van der Waals disjoining forces. The critical layer thickness is independent of the processing parameters, but presumably dependent only on material characteristics. In another system of poly(vinylidene fluoride-*co*-hexafluoropropylene) (PVDF-HFP)/polycarbonate (PC) with substantial rheological mismatch, at a nominal layer thickness below 160 nm, layers break up into microsheets and droplets triggered by viscoelastic differences between component melts (Figure 6), which dramatically alters the resulting dielectric properties [60]. Nevertheless, a clear mechanism responsible for layer breakup in the multilayered polymers still remains as an open question. More experimental and theoretical studies still need to be performed in order to better understand this phenomenon in the multilayer coextrusion process.

### 2.3. Interfacial Reaction

#### 2.3.1. Basic Theories

Considerable efforts have been dedicated to understanding the fundamental kinetics and mechanisms of interfacial reaction over the past 20 years. Theoretically, many studies are based on a planar interface in a simple model bilayer under static conditions, and the reaction kinetics reported can be generally categorized under two different mechanisms: a reaction-controlled mechanism and a diffusion-controlled mechanism. Fredrickson and Milner [61,62] studied the coupling reactions occurring at the interface between two symmetrical polymers (with the same degree of polymerization, *N_A_* = *N_B_*). The formation of copolymers, i.e., reactive coupling kinetics at polymer–polymer interfaces, was predicted to take place in three stages as follows. (i) At early times, the kinetics of the coupling reaction at the interface between reactive moieties controls the amount of copolymers at the interface, which (interfacial coverage) grows linearly with time. (ii) At intermediate times, the saturation of copolymers at the interface remains negligible, but a depletion hole of reactants builds up around the interface; the growth is dominated by the diffusion of the more dilute reactive species to the interface. (iii) For longer times, copolymers gradually form a copolymer layer at the interface. This interfacial copolymer layer generates a significant chemical potential barrier for unreacted homopolymers that strongly suppresses and controls the kinetics of the reaction by limiting the diffusion of reactive moieties to the interface for further reaction. O’Shaughnessy et al. [63,64] argued that the reaction over the first two steps should depend on the reactivity of the functional groups, especially for the reaction with weakly reactive pairs at the very beginning of the process. Nevertheless, they agreed on the third regime regarding the formation of an energy barrier resulting from the accumulation of copolymers at the interface. Generally speaking, the reaction-controlled mechanism is believed to be more appropriate in describing the short-time reaction for polymers with low reactivity. On the other hand, the diffusion-controlled mechanism is suitable in situations in which a reaction with high reactivity takes place over a longer time period. The transition from reaction-controlled kinetics to diffusion-controlled kinetics can be expected for reactions with high reactivity [65].

#### 2.3.2. Interfacial Morphology under Reaction

Theories on interfacial reaction are focused mainly on the reaction kinetics at very short times. Thus, researchers have shown that the accumulation of copolymers at the interface slows down the kinetics, because of the creation of an energy barrier preventing unreacted species from reaching the interface for further reaction. These studies were completed by experimental studies showing that this slower kinetics was spontaneously compensated, even in static conditions, by an acceleration due to the roughening of the interface. These interfacial fluctuations allow new reactive moieties to reach the interface and, therefore, the conversion rate to increase under certain conditions. In addition to an acceleration of the reaction kinetics, this interfacial roughening can generate small micelles in blend phases.

##### Reaction under Equilibrium Conditions

**Figure 7:**
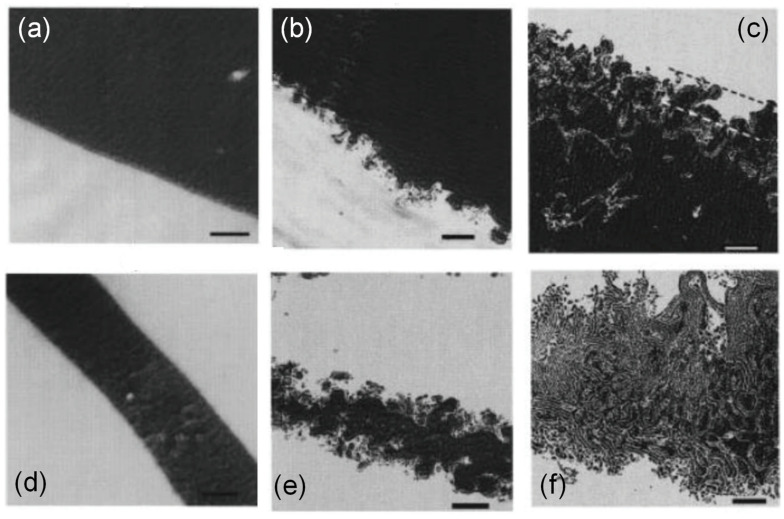
Representative morphologies of aliphatic amine-terminated polystyrene (PS-NH_2_)/anhydride-terminated poly(methyl methacrylate) (PMMA-ah) blends after static reaction times of (**a**) 0, (**b**) 20, and (**c**) 60 min at a large domain interface and after static reaction times of (**d**) 0, (**e**) 20, and (**f**) 60 min at a thin sheet interface at 200 °C. All scale bars are 500 nm. Dash lines in (**c**) approximately indicate the roughening zone. The magnitude of its width is roughly 500 nm. Reprinted with permission from Ref. [66]. Copyright (1999) American Chemical Society.

Macosko and coworkers performed a considerable number of studies on the interfacial reactions and morphological development at polymer–polymer interfaces under equilibrium conditions (without flows) [2]. For example, they examined the changes in the interfacial morphology upon the coupling reaction between aliphatic amine-terminated polystyrene (PS-NH_2_) and anhydride-terminated poly(methyl methacrylate) (PMMA-ah) [66]. A roughening interface can be observed after 20 min annealing as a result of the coupling reaction (Figure 7). Another interesting observation is that a lamellar microstructure appears at the interface of the thin domains after 1 h annealing, which indicates the formation of a PS-PMMA block copolymer. The interfacial roughening is explained by the large decrease in interfacial tension due to the creation of copolymers at the interface. The authors also supposed that local fluctuations might be induced by thermal fluctuations when block copolymer coverage at the interface decreases below the saturation level. This creates a new interfacial area that allows new reactive moieties to reach the interface and new copolymers to be formed. Interfacial instabilities continuously create a new interface in such a way that the interface becomes rough.

##### Morphology Development Revealed by Rheometry

**Figure 8:**
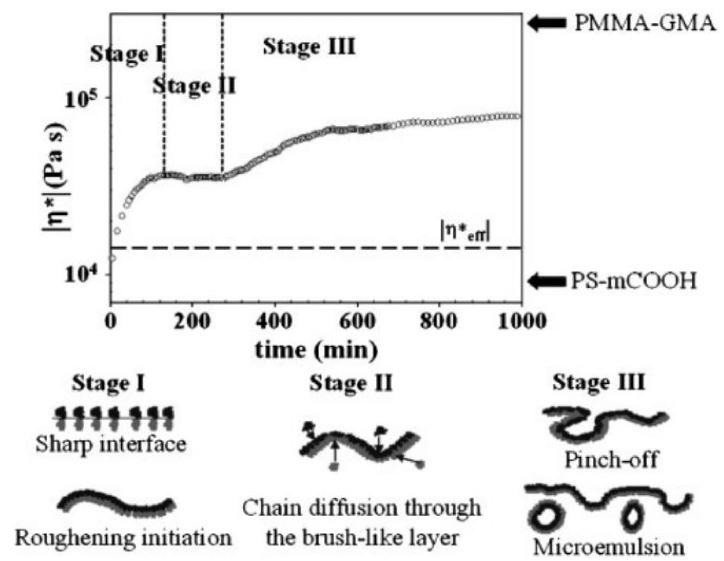
Complex viscosity as a function of reaction time for a monocarboxylated polystyrene (PS-mCOOH)/poly(methyl methacrylate-ran-glycidyl methacrylate) (PMMA-GMA) bilayer and schematic illustration describing variations of interfacial morphology. The dashed line is predicted by the reciprocal rule. Reprinted with permission from Ref. [67]. Copyright (2003) American Chemical Society.

Using a dynamic rheological measurement (small-amplitude oscillatory shear), the relationship between interfacial reactions and interfacial morphology development at a planar interface could be monitored the rheological responses [67,68,69]. Figure 8 presents the time evolution of the complex viscosity of a bilayer system based on end-functionalized monocarboxylated polystyrene (PS-mCOOH) and poly(methyl methacrylate-ran-glycidyl methacrylate) (PMMA-GMA) with reaction time measured at a strain of 0.005 and an angular frequency of 0.1 rad/s [67]. Based on the rheological responses and interfacial morphology captured by electron microscopes, the authors proposed three stages for the reaction: (i) in stage I, the sharp interface begins to undulate due to the creation of copolymers from the reaction at the interface; (ii) in stage II, the reactive moieties around the interface are totally consumed. New reactive moieties have to diffuse thorough the brush-like copolymer layer formed during stage I. The interface becomes corrugated; and (iii) in the final stage III, the reactive polymer chains penetrate again into the densely packed copolymer layers and allow the reaction to proceed further. The interfacial thickness and roughness increase. Further reaction even leads to the formation of some micelles (10–20 nm) and microemulsions (~100 nm) (which are micelles swollen with homopolymers) at the interfacial region. Apart from rheometry, the interfacial coupling reactions and morphology development at the planar interfaces of layered polymers could be also probed in situ by dielectric relaxation spectroscopy [13,70]. Using the dielectric molecular relaxation spectrum as a probe, the interfacial copolymer accumulation and resulting roughness could be captured and quantified, which agrees well with the rheological and morphological characterizations [70].

##### Effects of Oscillatory Shear Flow

Oscillatory shear amplitude and angular frequency also have a substantial influence on the interfacial reaction kinetics and morphology development [71]. A smaller strain amplitude (*γ*_0_) and angular frequency (*ω*) were found to enhance the extent of reaction and the generation of an interphase. By contrast, at larger strains and angular frequencies, oscillatory shearing inhibits the diffusion of polymer chains to the interface and thus also inhibits reactions therein. In addition, a large strain can generate alternating layers of PS-mCOOH/PMMA-*graft*-PS copolymer, which act as a barrier for the diffusion of reactive chains to the interface, thereby restricting interfacial reactions. On the other hand, a higher *ω* can break the interface and generate a multilayer of graft copolymer. In this situation, even though a lower *ω* is applied again, further reaction does not occur; this inhibition, therefore, becomes a permanent obstacle to further interfacial reactions. Additionally, it is observed that the perpendicular shear force greatly enhances the roughness of the interface as compared with parallel shear force applied to a reactive bilayer [72]. Here, the perpendicular shearing applied to the reactive bilayer also leads to the formation of microemulsions that could markedly stabilize the interfacial morphology.

#### 2.3.3. Interfacial Reaction in Multilayer Coextrusion

**Figure 9:**
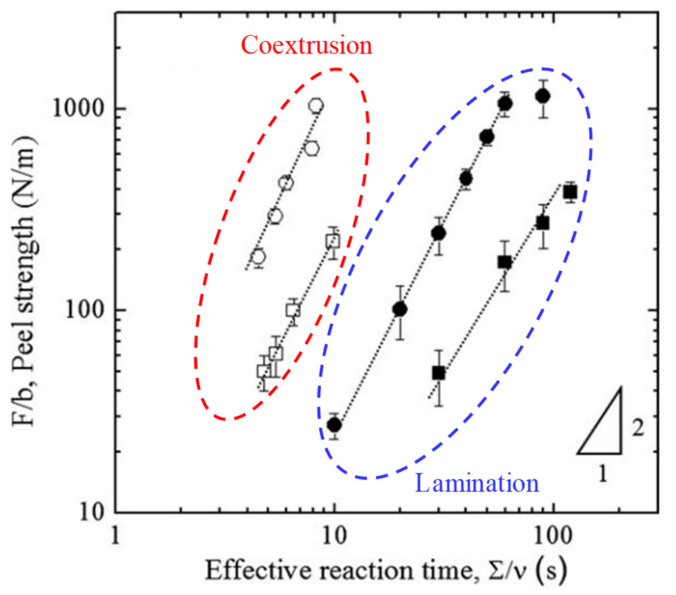
Comparison for peel strength of polyurethane (PU) and PE-amine groups (NHR) with different NHR functionalities produced from coextrusion and lamination. The circles indicate NHR functionality of 3 wt%, and squares represent NHR functionality of 1 wt%. Reproduced with permission from Ref. [79]. Copyright (2011) John Wiley and Sons.

Macosko and coworkers [73] investigated the interfacial coupling reaction during coextrusion with a 640-layer PS-NH_2_/anthracene-labeled anhydride-terminal PMMA (PMMA-anh-anth) system prepared by multilayer coextrusion. They found that a significant amount of PS-b-PMMA copolymers are formed during coextrusion, such that the interfaces were almost saturated with the block copolymers formed in situ. The interface became roughened, and interfacial emulsification could be observed due to high block copolymer coverage. Coextrusion processing significantly accelerated the coupling reaction of PS-NH_2_/PMMA-anh and reduced the reaction time by more than three orders of magnitude as compared with that under quiescent annealing. The external flow under coextrusion could overcome the combined effects of high surface energy of the functional groups and slow diffusion, increasing the functional group concentration in the interfaces. In particular, the extensional deformation could be more important than shear in accelerating coupling. Flow-accelerated interfacial reaction kinetics were also noticed in other layered systems [74,75]. In a recent study, the compressive flow in coextrusion was demonstrated to play a crucial role in accelerating the coupling reaction [76]. Strikingly, coextrusion with compressive flow resulted in a reaction rate two orders of magnitude faster than that without compressive flow and enabled stronger adhesion. Besides, mechanical properties of coextruded multilayers with in-situ compatibilization reactions are significantly enhanced [7,77,78]. As illustrated by Figure 9, coextruded layers of polyurethane (PU) and functional polyethylene (PE) with amine groups (NHR) exhibit dramatically the higher peel strength at a shorter reaction time in comparison with these produced from lamination process [79]. This acceleration was attributed to the combined extensional and compressive flows in coextrusion that overcome the diffusion barrier at the interface and forcing reactive species to penetrate the interface. The interfacial reinforcement in the real micro-/nanolayer coextrusion show a strong dependence on the reaction time and the number of layers [13,80]. The resulting large enhancement in interfacial stress with the number of layers, characterized by an extensional strain hardening behavior, is attributed to the increased density of copolymers at the interfaces.

**Figure 10:**
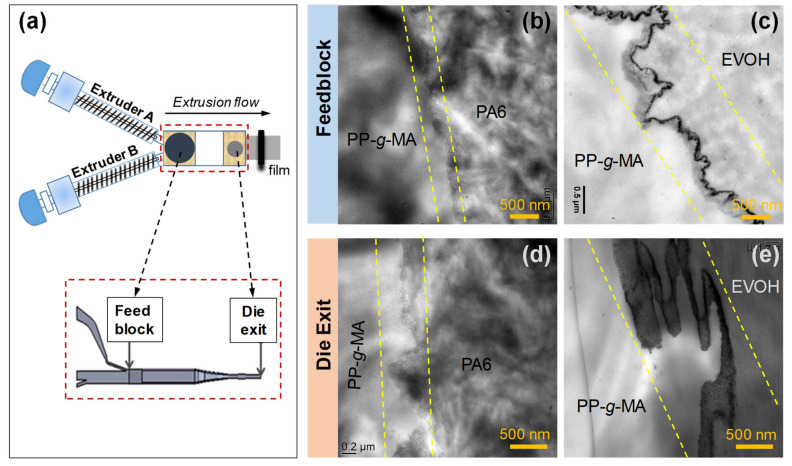
(**a**) Schematic of the coextrusion device with feedblock and die exit system. TEM micrographs of interfacial morphology for (**b**) PP-*g*-MA/PA6 and (**c**) PP-*g*-MA/EVOH bilayers quenched after leaving the feedblock and for (**d**) PP-*g*-MA/PA6 and (**e**) PP-*g*-MA/EVOH bilayers quenched after leaving the die exit. The area sandwiched between parallel dashed lines in (**b**–**e**) indicates the interfacial regions. Reprinted with permission from Ref. [83]. Copyright (2020) American Chemical Society.

Lamnawar and coworkers [81,82,83] prepared multilayer films of PA6/PP and EVOH/PP systems with maleic anhydride-grafted polypropylene (PP-*g*-AM) as a tie layer using reactive coextrusion. The macromolecular architecture of the copolymers formed at the interfaces critically influences the interfacial morphology evolution, viscoelasticity and processability of layered systems. Specifically, the interface in the pure grafting case of PA6/PP-*g*-AM bilayer is relatively flat, while the interface in EVOH/PP-*g*-AM bilayer is irregular and rough due to the presence of mixed grafting and cross-linking reactions. Particularly, under real coextrusion flows, with these copolymers of complex architecture, interfacial roughness and irregularities are further enlarged in the die exit as compared to those in the feedblock due to the accelerated reaction kinetics (Figure 10). Additionally, the higher surface density of the EVOH-*co*-PP-*g*-MA copolymers generated at the interfaces further leads to an optical grainy defect and a loss of transparency in the resulting multilayer films [82,84].

### 2.4. Interfacial Confinement in Micro-/Nanolayer Coextrusion

Confinement of polymeric systems at the nanometer scale has revealed that under constraint, many properties including crystallization, physical aging, permittivity, and glass transition deviate from bulk material characteristics [85,86,87]. Interestingly, confinement phenomena also occur in the multilayer coextruded polymers, especially for nanolayered films with thousands of layers in which all layers have nanometric thicknesses. The nanoconfinement effect plays an important role in tailoring macroscopic properties of as-coextruded films [24,88,89]. In this section, we introduce the effects of interfacial geometrical confinement on crystallization and glass transitions in multilayered films produced by coextrusion.

#### 2.4.1. Confined Crystallization

**Figure 11:**
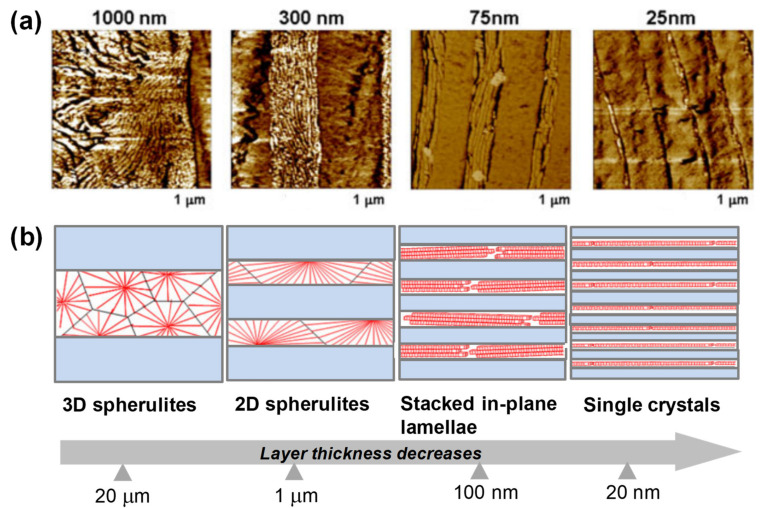
(**a**) AFM phase images of cross-sections of PS/poly(ethylene oxide) (PEO) layered films with 1000, 300, 75, and 25 nm thick PEO layers from left to right. (**b**) Schematic morphology evolution of PEO confined layers as the layer thickness is reduced from the microscale to the nanoscale. Reprinted with permission from Ref. [25]. Copyright (2012) Cambridge University Press.

Geometrical/spatial confinement in multilayer coextrusion remarkably alters the crystallization behaviors and morphology within the confined crystalline polymer layers [25]. When layer thickness decreases, the crystalline morphology is gradually changed from a three-dimensional (3D) spherulite morphology into one-dimensional (1D) lamellar morphology. The confined crystallization in multilayer coextrusion has been observed in many systems with semicrystalline polymers as confined materials, including polypropylene (PP) [90,91], polyethylene (PE) [92,93], poly(ε-caprolactone) (PCL) [94], and poly(ethylene oxide) (PEO) [23,95], typically confined by amorphous rigid polymers with the higher glass transition temperatures (e.g., PS) (i.e., confining materials). When the layer thickness decreases below the diameter of spherulites (100 μm), the lamellae organize into flattened/compressed spherulites as discoids, and the lamellae usually have a preferred in-plane orientation, such as edge-on morphology. For example, PEO confined by PS forms in-plane crystals with an improved orientation by reducing the layer thickness and even crystallizes into a single, in-plane, high aspect ratio lamellae crystal when layer thickness reaches the scale of lamellae thickness (Figure 11) [25,96]. With further reduction in the layer thickness, the lamellae display edge-on morphology. In addition, a substantial reduction in permeability (by more than two orders of magnitude) has been noticed under confinement due to increased tortuosity of the diffusion pathway through the highly oriented lamellae [96]. With other crystalline polymers such as PVDF, geometrical confinement can induce other types of crystal morphologies and lamellar orientation [25,97,98]. It has been generally accepted that this confinement in the PVDF-containing multilayers greatly affects the resulting dielectric properties when used as capacitors [99,100,101].

#### 2.4.2. Confined Glass Transition Dynamics

**Figure 12:**
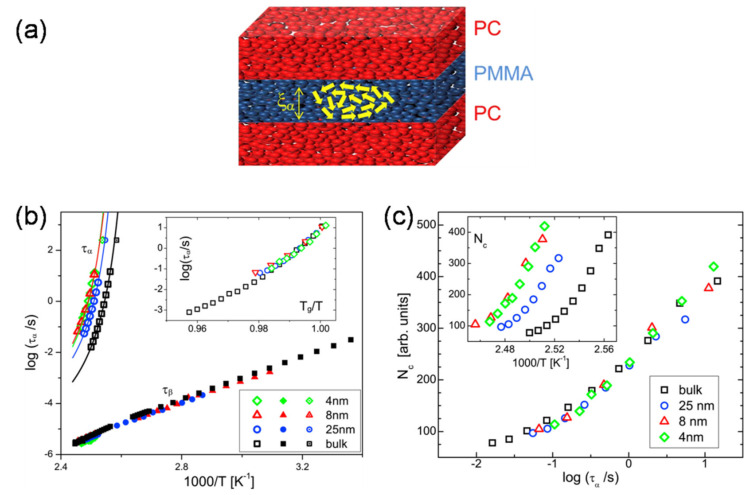
(**a**) Comparison of confinement length scale with correction length of confining PMMA in PC/PMMA multilayers. (**b**) Activation plots of segmental α-relaxation time (*τ*_α_) for PC/PMMA multilayers and bulk PMMA. (**c**) Number of correlated units (*N*_c_) versus the α-relaxation time for bulk and nanoconfined PMMA samples. Notwithstanding the large changes in *N*_c_ due to confinement (inset), the dependence of *N*_c_ on *τ*_α_ is the same as for the bulk PMMA. Reproduced with permission from Ref. [104]. Copyright (2016) Elsevier.

Confinement at the layer–layer interfaces also influences the glass transitions of as-coextruded multilayer films [102]. For instance, a merging in glass transition temperatures (*T_g_*) with increasing the number of layers was ever observed in PC/PMMA multilayer films, especially when the individual layer thicknesses were below 10 nm [32,33]. In particular, the degree of confinement depends on the layer thickness and the correction length scale of the confined layer materials. Delbreilh and coworkers [103] investigated molecular mobility at the glass transition evolution in PC/PMMA multilayered films from micro- to nanoscale, including the glass transition temperature (*T_g_*) and cooperatively rearranging region (CRR) size. The molecular mobility in each polymer is found to be altered in entirely different ways as the layer thickness becomes thinner than 125 nm, whereby the constituent polymers exist as two-dimensional layers under these conditions. PC exhibits a drastic decrease in cooperativity volume at the glass transition due to the confinement effect associated with conformational changes in the macromolecular chain. By contrast, slight modifications were observed for PMMA, due to the weaker intermolecular interactions in the main chains compared to those of PC. However, in a recent study with PC/PMMA multilayered films with layer thickness as thin as 4 nm, Casalini et al. recently reported an absence of geometric confinement effects on dynamic correlation (CCR size) of PMMA (i.e., the same as the bulk), especially when the confinement length scale (layer thickness) approaches or is less than the correlation length scale (*ξ*) (Figure 12a) [104]. Neither the fragility nor the breadth of the relaxation dispersion was affected by the geometric confinement therein (Figure 12b,c). More significantly, the dynamic correlation volume/length representing the cooperativity of the dynamics was also unaffected. Instead, the increase in the local segmental relaxation time and glass transition temperature of PMMA with decreasing layer thickness was primarily attributed to its intermixing with the high-*T_g_* component (PC) at the extended interfacial region.

## 3. Conclusions

In conclusion, this paper presents a systematic review of the interfacial phenomena involved in multi-micro-/nanolayered polymer coextrusion, from the dual viewpoints of fundamental science and engineering. These phenomena, including interlayer diffusion, interlayer reaction, interfacial instabilities, and interfacial geometrical confinement, frequently occur at the layer–layer interfaces of multilayered polymers produced by multilayer coextrusion processing. The origin and basic theories of these interfacial phenomena are explained, along with the way in which they can affect microstructural development and the resulting macroscopic properties. In particular, these interfacial phenomena play a significant role in determining the interlayer adhesive strength, glass transitions, mechanical, transport, and other physicochemical properties of coextruded multilayer polymers. These phenomena occurring in multilayer systems generally show strong dependences on the layer compositions, layer thicknesses, the number of layers, processing conditions (i.e., flow fields and temperature), and the inherent characteristics of constituent polymers as well. Different approaches to investigating such effects have also been summarized, including numerical methods, microscopy, rheology, dielectric spectroscopy, energy-dispersive X-ray analysis, thermal and dynamic analyses, etc. More intensive work should be carried out in both the academia and industry to further clarify exactly what occurs at layer–layer interfaces with a view toward the scaling-up of multilayer coextrusion. Hopefully, this paper will promote a better understanding of the interfacial phenomena of multicomponent polymers and facilitate the establishment of a processing-structure-property relationship. In addition, this review provides some guidelines for controlling the interfaces and microstructure in multilayered polymer systems intended for use in advanced applications.

## Data Availability

Not applicable.
